# 5Z‐7‐Oxozaenol attenuates cuprizone‐induced demyelination in mice through microglia polarization regulation

**DOI:** 10.1002/brb3.3487

**Published:** 2024-04-22

**Authors:** Shiyu Chen, Siyao Liu, Yalun Huang, Shiwen Huang, Wanzhou Zhang, Huifang Xie, Lingli Lu

**Affiliations:** ^1^ Department of Neurology Zhujiang Hospital, Southern Medical University Guangzhou China; ^2^ Department of General Practice Zhujiang Hospital, Southern Medical University Guangzhou China

**Keywords:** 5Z‐7‐Oxozeaenol, demyelination, microglia polarization

## Abstract

**Introduction:**

Demyelination is a key factor in axonal degeneration and neural loss, leading to disability in multiple sclerosis (MS) patients. Transforming growth factor beta activated kinase 1 (TAK1) is a critical molecule involved in immune and inflammatory signaling pathways. Knockout of microglia TAK1 can inhibit autoimmune inflammation of the brain and spinal cord and improve the outcome of MS. However, it is unclear whether inhibiting TAK1 can alleviate demyelination.

**Methods:**

Eight‐week‐old male c57bl/6j mice were randomly divided into five groups: (a) the control group, (b) the group treated with cuprizone (CPZ) only, (c) the group treated with 5Z‐7‐Oxozaenol (OZ) only, and (d) the group treated with both cuprizone and 15 μg/30 μg OZ. Demyelination in the mice of this study was induced by administration of CPZ (ig) at a daily dose of 400 mg/kg for consecutive 5 weeks. OZ was intraperitoneally administered at mentioned doses twice a week, starting from week 3 after beginning cuprizone treatment. Histology, rotarod test, grasping test, pole test, Western blot, RT‐PCR, and ELISA were used to evaluate corpus callosum demyelination, behavioral impairment, oligodendrocyte differentiation, TAK1 signaling pathway expression, microglia, and related cytokines.

**Results:**

Our results demonstrated that OZ protected against myelin loss and behavior impairment caused by CPZ. Additionally, OZ rescued the loss of oligodendrocytes in CPZ‐induced mice. OZ inhibited the activation of JNK, p65, and p38 pathways, transformed M1 polarized microglia into M2 phenotype, and increased brain‐derived neurotrophic factor (BDNF) expression to attenuate demyelination in CPZ‐treated mice. Furthermore, OZ reduced the expression of proinflammatory cytokines and increases anti‐inflammatory cytokines in CPZ‐treated mice.

**Conclusion:**

These findings suggest that inhibiting TAK1 may be an effective approach for treating demyelinating diseases.

## Graphical abstract

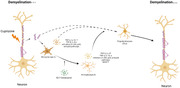

## 1 INTRODUCTION

Multiple sclerosis (MS) is a chronic autoimmune and inflammatory demyelinating disease of central nervous system (CNS) that primarily affects young individuals worldwide (Marcus, 2022). The exact pathology and etiology of MS remain unclear, and current immunomodulation therapy for MS mainly targets CNS inflammation, lacking the ability to regenerate myelin and prevent disease progression (Olek et al., 2021). Demyelination of the brain and spinal cord is major characteristic of MS, resulting in impaired generation of oligodendrocytes from oligodendrocyte precursor cells (OPCs). This persistent demyelination, along with myelin debris accumulation and axonal damage, leads to neurological disability (Dulamea, 2017). Therefore, efficient clearance of myelin debris by phagocytic cells is crucial to eliminate inhibitory signals that interfere with OPC activation, recruitment, and differentiation into myelinating mature oligodendrocytes (Gudi et al., 2014).

Microglia and infiltrating monocytes/macrophages play dual roles in MS lesions. They can cause damage to the myelin sheath and enlarge of the lesion, but they can also play a protective role by clearing myelin debris, reducing inflammation, and secreting regenerative factors that promote myelin regeneration (Voet et al., 2019). Activated microglia secrete proinflammatory cytokines, leading to further damage, or promote tissue repair and synaptic remodeling through anti‐inflammatory factors and neurotrophic factors, depending on the duration and intensity of stimulation (Eyo & Wu, 2019; Ma et al., 2017; Wang et al., 2020). Inhibiting TAK1 in microglia has been shown to have therapeutic effects in experimental autoimmune encephalomyelitis (EAE) mice, an animal model widely used for immune studies in MS, by reducing the expression of inflammatory mediators and related chemokines (Goldmann et al., 2013). This suggests that inhibiting TAK1 may be a potential therapeutic strategy for MS.

5Z‐7‐Oxozeaenol (OZ), a classic irreversible inhibitor of TAK1, has been shown to attenuate inflammation and tissue damage in various conditions, including ischemic stroke, Alzheimer's disease, dorsal root ganglion neurons, and EAE mice (Chen et al., 2015; Liddelow et al., 2017; Wang et al., 2020). Our previous research has demonstrated that inhibiting TAK1 by OZ can reduce the inflammatory response and neurological deficits in the CNS of EAE mice (Lu et al., 2017). Since compounds that have therapeutic effects on EAE may not necessarily be effective in MS patients, whose neurological disability is mainly caused by demyelination. Cuprizone (CPZ) is commonly used to induce toxic demyelination in animal models. This allows researchers to selectively study the demyelination and remyelination processes, which are similar to those observed in MS.

Thereby, the present study aimed to investigate the outcome and possible underlying mechanisms of OZ on MS using CPZ model. We hypothesis that OZ can alleviate behavioral symptoms and myelin loss. We propose a mechanism of action involving the regulation of microglia‐induced neuroinflammation and the induction of M2 type microglia, as well as prevention the loss of oligodendrocytes. Our findings suggest that TAK1 inhibition may represent a promising approach for the treatment of demyelinating diseases.

## 2 MATERIALS AND METHODS

### 2.1 Reagents, antibodies, and animals

The sources and identifiers of reagents and antibodies used in the study are listed in Table 1.

**TABLE 1:** Key resources used in this study.

Reagent type	Designation	Source	Identifiers	Additional information	Dilution	RRID
Compound	Cuprizone	Sigma‐Aldrich	14690	MO, USA	N/A	N/A
Compound	5Z‐7‐Oxozeaenol	MedChemExpress	HY‐12686	Shanghai, CHN	N/A	N/A
Antibody	anti‐TAK1	Affinity Biosciences	AF7616	OH, USA	1:500	AB_2843980
Antibody	Phospho‐TAK1	Affinity Biosciences	AF4379	OH, USA	1:500	AB_2844444
Antibody	NF‐kB p65	Affinity Biosciences	AF5006	OH, USA	1:500	AB_2834847
Antibody	Phospho‐NF‐kB p65	Affinity Biosciences	Ser536	OH, USA	1:500	AB_2834435
Antibody	anti‐JNK1/2/3 (Phospho‐Thr183+Tyr185)	EnoGene	E1A0500A	Nanjing, CHN	1:500	AB_3094644
Antibody	JNK1/2/3 (Ab‐183/185)	EnoGene	E1A0500B	Nanjing, CHN	1:500	AB_3094645
Antibody	p38 MAPK (Phospho‐Thr180)	EnoGene	E1A7178A	Nanjing, CHN	1:500	AB_3094646
Antibody	P38 MAPK (Ab‐180)	EnoGene	E1A7178B	Nanjing, CHN	1:500	AB_3094647
Antibody	ionized calcium‐binding adaptor molecule 1 (Iba‐1)	WAKO	019‐19741	Japan	1:1000	AB_839504
Antibody	Arginase‐1	Cell Signaling Technology	93668T	MA, USA	1:500	AB_2800207
Antibody	anti‐iNOS	Proteintech Group	18985‐1‐AP	IL, USA	1:500	AB_2782960
Antibody	BDNF	Proteintech Group	28205–1	IL, USA	1:500	AB_2818984
Antibody	GAPDH	Proteintech Group	60004–1	IL, USA	1:20000	AB_2107436
Antibody	anti‐APC	Abcam	ab16794	MA, USA	1:200	AB_443473
Antibody	Olig2	Millipore	MABN50	MO, USA	1:500	AB_10807410
Antibody	Horseradish peroxidase (HRP)‐labeled goat anti‐rabbit IgG	Beyotime Biotechnology	A0208	Shanghai, CHN	1:1000	AB_2892644
Antibody	HRP‐labeled goat anti‐mouse IgG	Beyotime Biotechnology	A0216	Shanghai, CHN	1:1000	AB_2860575
Antibody	alexa Fluor 488 goat anti‐rabbit IgG (H + L)	Beyotime Biotechnology	A0423	Shanghai, CHN	1:500	AB_2891323
Antibody	alexa Fluor 555 donkey anti‐mouse IgG (H + L)	Beyotime Biotechnology	A0460	Shanghai, CHN	1:500	AB_2890133

Male C57BL/6 mice (*n* = 50), aged 8 weeks (weight, 22 ± 1 g), were obtained from Sijiajingda Bio‐Tech Co., Ltd. (Guangzhou, China). Sample size calculations were performed using an online sample size calculator (https://clincalc.com/stats/samplesize.aspx). The allocation of mice in each group was randomized and blinded. The mice were housed in an ambient temperature environment (22 ± 2°C) under a 12‐h dark/light cycle ((07:00, lights on; 19:00, lights off), with free access to water and food. All animal husbandry and experiments conducted in this study were approved by the Institutional Animal Care and Use Committee (IACUC) of Zhujiang Hospital, Southern Medical University, under protocol # LAEC‐2021‐052.

### 2.2 Cuprizone treatment

After adaptation for 1 week, mice (*n*  =  50) were randomly divided into five equal‐sized testing groups: control under DMSO treatment (Ctrl + DMSO), control under 5Z‐7‐Oxozeaenol treatment at a dose of 30 μg (Ctrl + 30 μg OZ), cuprizone under DMSO treatment (CPZ + DMSO), and cuprizone groups under treatment with OZ at a dose of 15 μg (CPZ + 15 μg OZ) or 30 μg (CPZ + 30 μg OZ). To administer cuprizone through oral feeding via gavage needle, cuprizone (Merck Millipore, Germany) was mixed with 0.5% carboxymethyl cellulose (CMC) and stirred to produce a consistent suspension. The control group received only the 0.5% CMC. Demyelination in the mice of this study was induced by administration of CPZ (ig) at a daily dose of 400 mg/kg for consecutive 5 weeks (Zhen et al., 2017). OZ was intraperitoneally administered at mentioned doses twice a week, starting from week 3 after beginning cuprizone challenge until the end of week 5 post‐cuprizone.

### 2.3 Behavioral tests

#### 2.3.1 Rotarod test

The rotarod test was implemented as described previously (Mihai et al., 2021). Mice were placed on a Rotarod (Panlab LE8505, USA). Each mouse was trained at 10 rpm for 3 days prior to the testing day. During the test, the rotarod speed was increased from 4 to 30 rpm in 300 s, and the time to the first fall off the drum was recorded. Each mouse underwent three experiments with a 15‐min interval.

### 2.4 Grasping test

The muscle grip strength was assessed using the grasping test as described previously (Brooks & Dunnett, 2009). Mice were hanged by their forepaws on a horizontal wire (diameter, 2 mm; 40 cm from the ground) for 10 s. During this time, the grasping score was recorded as follows: grasping the wire with both hind legs achieved a score of 3; grasping the wire with only one hind leg achieved a score of 2; grasping the wire with either hind leg achieved a score of 1; and dropping achieved a score of 0. The final score was determined by the average score of the three trials.

#### 2.4.1 Pole test

This method was carried out as described in our previous work (Khaledi et al., 2021). Mice were pretrained with the pole (height, 75 cm; diameter, 10 mm; wrapped with gauze) three times to ensure that all mice would climb down smoothly when they were placed head up on the top of a pole. The total time it took the mice to climb down the pole was recorded.

### 2.5 Luxol fast blue (LFB) staining

Mouse brains were embedded and cut into 15‐μm sections, followed by deparaffinization. The sections were then rehydrated in a solution of LFB (0.01% LFB in 95% ethanol) and incubated overnight at 56°C. After washing in 95% and 75% ethanol and removing excess stain, the sections were differentiated in lithium carbonate solution for 15 s, followed by washing in distilled water. Subsequently, the sections were dehydrated and fixed. Images were scanned using PANNORAMIC Digital Slide Scanners (3DHISTECH Ltd, Hungary). Quantification of LFB staining was performed within the corpus callosum zone of three mice per group using the image analysis system Image‐Pro Plus 6.0 (IPP 6.0, Media Cybernetics, Bethesda, MD, USA). The LFB intensity was measured and normalized to the corresponding area to obtain the final calculated results.

### 2.6 Western blotting

Mouse brain tissue was sliced along the cerebral sulcus, and the corpus callosum tissue was carefully separated. The corpus callosum tissues were collected, and total protein was extracted using RIPA Lysis Buffer (Beyotime Biotechnology). Protein concentration was determined using a Bicinchoninic Acid (BCA) Protein Assay Kit (Beyotime Biotechnology). The samples were then separated by SDS‐PAGE and transferred to PVDF membranes. The membranes were blocked with 5% bovine serum albumin (BSA) and incubated with primary antibody. After incubation with secondary antibody, chemiluminescence was visualized using the Nice Alliance Q9 system (UVITEC, Cambridge, United Kingdom). The optical density of the bands was analyzed using ImageJ software. Grayscale values were obtained and subsequently normalized to the expression level of the housekeeping protein GAPDH. The ratio of grayscale values was then used to determine the relative protein expression level.

### 2.7 Immunofluorescence assays

Mouse brains were embedded and cut into 30‐μm sections using a freezing microtome (Leica). The sections were incubated overnight at 4°C with corresponding primary antibodies. After incubation with fluorescent‐labeled secondary antibodies and staining the nuclei with DAPI, the brain slices were scanned using an ECLIPSE Ti2 inverted microscope (Nikon) with consistent acquisition parameters for each immunostaining. The acquired images were then subjected to quantitative analysis of immunofluorescence staining using the Image‐Pro Plus 6.0 software (IPP 6.0, Media Cybernetics, Bethesda, MD, USA). An investigator who was blinded to the experimental groups performed the acquisition of images and quantitative analysis of immunofluorescence staining. The immunofluorescence density was measured and analyzed using the Image‐Pro Plus 6.0 software. The counts of CC1^+^‐, Olig2^+^‐, and Iba1^+^‐ cells were conducted in three specific fields within the corpus callosum zone of three mice per group. Additionally, microglia morphology was quantified based on a previous report (Young & Morrison, 2018). This involved converting the fluorescence images into binary images and using ImageJ software for skeletonization to collect the number of endpoints and process lengths per frame, which were used to measure the morphology of microglia.

### 2.8 Quantitative RT‐PCR

The mRNA expression levels of inflammatory cytokines in the cerebral cortex region were measured. Total RNA was extracted from the tissues using Trizol reagent (Sangon Biotech, Shanghai, China) following the manufacturer's protocol. The quality and concentration of RNA was assessed by measuring the absorbance at 260 and 280 nm using a spectrophotometer (NanoDrop 1000, NanoDrop Technologies, LLC, USA). Genomic DNA was removed using gDNA Clean Reagent (Accurate Biology, Hunan, China). The RNA was reverse transcribed into complementary DNA (cDNA) using Evo M‐MLV RT Kit (Accurate Biology). qPCR was performed with SYBR Green Pro Taq (Accurate Biology) on Applied Biosystems QuantStudio 6&7 (Thermo Fisher Scientific, Waltham, USA). The PCR reaction was subjected to 40 cycles under the reaction conditions as follows: Predenaturation at 96°C for 4 min, denaturation at 95°C for 5 s; annealing and extension at 60°C for 30 s. mRNA expression levels relative to GAPDH were calculated using the 2–△△Ct method. Data are expressed as relative amount of the target genes to GAPDH, respectively. The primer sequences are provided in Table 2.

**TABLE 2:** RT‐PCR primers sequences.

Gene	Forward primer	Reverse primer
GAPDH	ACGGGAAGCTCACTGGCATGGCCTT	CATGAGGTCCACCACCCTGTTGCTG
IL‐1	AATGCCACCTTTTGACAGTGAT	TGCTGCGAGATTTGAAGCTG
TNF‐α	CTTGTTGCCTCCTCTTTTGCTTA	CTTTATTTCTCTCAATGACCCGTAG
IL‐4	GGTCTCAACCCCCAGCTAGT	GCCGATGATCTCTCTCAAGTGAT
TGF‐β	TGCGCTTGCAGAGATTAAAA	CGTCAAAAGACAGCCACTCA

GAPDH, glyceraldehyde‐3‐phosphate dehydrogenase; IL‐1, interleukin 1; TNF‐α, tumor necrosis Factor‐α; IL‐4, interleukin 4; TGF‐β, transforming growth factor‐β.

### 2.9 Enzyme‐linked immunosorbent assay

Enzyme‐linked immunosorbent assay (ELISA) kits from Wuhan Servicebio Technology Co., Ltd. were employed to measure the concentrations of TNF‐α, IL‐1β, TGF‐β, and IL‐4 in the mouse cerebral cortex. Briefly, samples were homogenized in ice‐cold PBS, and the resulting homogenates were centrifuged at 3000 rpm for 10 min at 4°C to obtain the supernatants. The protein concentrations in the supernatants were determined using a BCA assay. Subsequently, the supernatants were incubated with antibody‐coated plates specific to the target cytokines, followed by incubation with HRP‐labeled secondary antibodies at 37°C for 1 h. Afterward, chromogenic agents A and B were added and incubated at 37°C for 15 min. The optical density (OD) values were measured at 450 nm using an Enzyme Label Detector (BioTek, USA). The results were expressed as picograms (pg) of cytokine per milligram (mg) of protein.

### 2.10 Statistical analysis

All data are presented as the mean ± standard error of the mean (SEM). Statistical analysis of multiple comparisons was performed using one‐way analysis of variance (ANOVA), followed by Tukey's post hoc test. All statistical analyses were conducted using SPSS 24.0 to assess the correlations between different experiments. The data were presented in histogram format using GraphPad Prism 8.0 software. Differences with a *p*‐value < .05 were considered statistically significant.

## 3 RESULTS

### 3.1 OZ treatment improves motor deficits and protects against myelin loss in demyelinated mice

**FIGURE 1:**
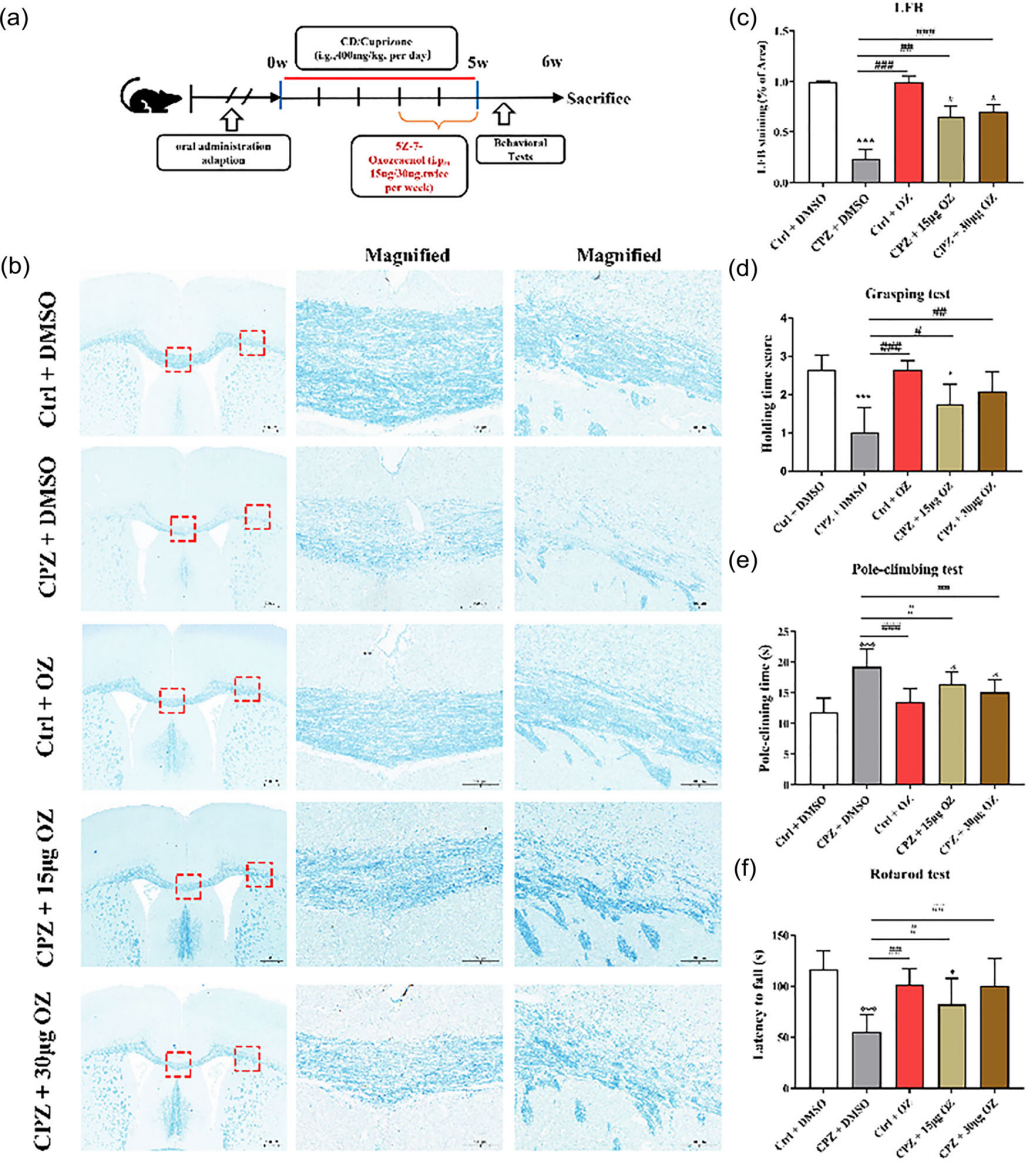
OZ treatment improved motor deficits and protected against myelin loss in CPZ mice. (a) Experimental design for OZ treatment in CPZ‐induced demyelinated mice. (b) Representative Luxol fast blue (LFB) staining of corpus callosum in Ctrl + DMSO, CPZ + DMSO, Ctrl + OZ, CPZ + 15 μg OZ and CPZ + 30 μg OZ groups. (c) Quantification of demyelination in the corpus callosum (*n* = 3). (d) Grip strength was assessed using the grasping test (*n* = 10). (e) Motor coordination was assessed using the pole‐climbing test (*n* = 10). (f) Motor coordination was assessed using the rotarod test (*n* = 10). Data are presented as the mean ± SEM. ^***^ *p* < .001, ^**^ *p* < .01, ^*^ *p* < .05 vs. the Ctrl + DMSO group. ^###^ *p* < .001 ^##^ *p* < .01, ^#^ *p* < .05 vs. the CPZ + DMSO group. One‐way ANOVA with Tukey's post hoc analysis was used for comparison among multiple groups.

To evaluate the neuroprotective effects of Tak1‐inhibitor OZ, we utilized a CPZ‐induced demyelinated mouse model. OZ treatment was initiated after 3 weeks of CPZ induction (Figure 1a). The myelin sheath condition was assessed by LFB staining of the corpus callosum. Compared to the CPZ + DMSO group, treatment with 15 and 30 μg of OZ significantly increased the myelination intensity in the corpus callosum in the CPZ + 15 μg OZ and CPZ + 30 μg OZ groups (*F* = 58.036, *p *< .001). There were no differences in myelination intensity between the Ctrl + DMSO and Ctrl + OZ groups (Figure 1b **and** c). The loss of the myelin sheath in the corpus callosum can result in impaired motor neurological function in mice. The severity of the neurological deficit increases with the extent of myelin loss. In the grasping test, the holding time score decreased in the CPZ + DMSO group compared to the Ctrl + DMSO group. However, OZ treatment at 15 and 30 μg significantly increased the holding time score in CPZ‐induced mice (*F* = 19.195, *p* < .001) (Figure 1d). Similarly, in the pole‐climbing test, the CPZ + 15 μg OZ and CPZ + 30 μg OZ groups showed decreased pole‐climbing time compared with the CPZ + DMSO group (*F* = 14.698, *p* < .001) (Figure 1e). In the rotarod test, CPZ + DMSO group had lower latencies to fall than the Ctrl + DMSO and Ctrl + OZ groups. However, treatment with 15 and 30 μg of OZ increased the latency time to fall in CPZ mice compared with the CPZ + DMSO group (*F* = 12.574, *p* < .001) (Figure 1f). No significant differences were observed in the behavioral tests between the Ctrl + DMSO and Ctrl + OZ groups (Figure 1d–f). Interestingly, mice treated with CPZ did not show significant differences in motor behavior at the 15 μg and 30 μg doses of OZ. These results suggest that OZ treatment can ameliorate the motor balance and coordination ability impairment caused by CPZ.

### 3.2 OZ rescued the loss of oligodendrocytes in CPZ‐induced mice

**FIGURE 2:**
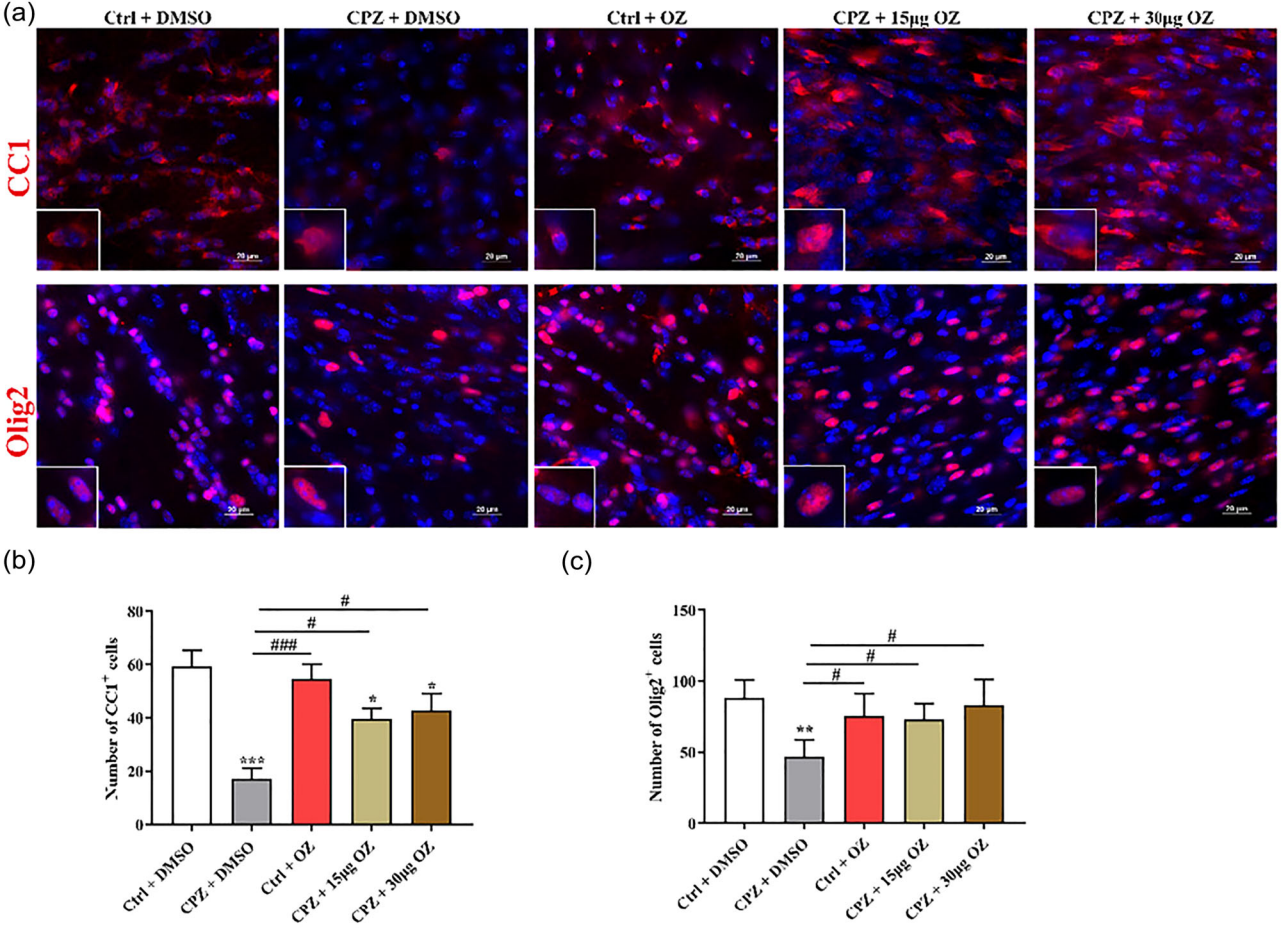
OZ rescued the loss of oligodendrocytes in CPZ‐induced mice. (a) Representative immunofluorescence images of CC1 and Olig2 staining in the corpus callosum of control or CPZ‐treated mice with or without OZ treatment (*n* = 4). Scale bar = 20 μm. (b, c) Quantification of the CC1^+^ and Olig2^+^ cells expression in the five groups; Data are presented as the mean ± SEM. ^***^ *p* < .001, ^**^ *p* < .01, ^*^ *p* < .05 vs. the Ctrl + DMSO group. ^###^ *p* < .001, ^##^ *p* < .01, ^#^ *p* < .05 vs. the CPZ + DMSO group. One‐way ANOVA with Tukey's post hoc analysis was used for comparison among multiple groups.

To confirm the role of OZ in oligodendrocyte lineage development and myelination, we use specific markers Olig2 (oligodendrocytes) and CC1 (mature oligodendrocytes). As shown in Figure 2a and b, CPZ significantly reduced the number of CC1^+^ cells in the corpus callosum of mice. Compared to CPZ + DMSO group, there were more CC1^+^ cells in CPZ + 15 μg OZ and CPZ + 30 μg OZ groups (*F* = 28.909, *p* < .001). Compared to Ctrl + DMSO group, CPZ also decreased the number of Olig2^+^ cells in the corpus callosum of mice, while treatment with 15 and 30 μg OZ treatment increased the number of Olig2^+^ cells (*F* = 3.690, *p* < .05) (Figure 2a and c). There was no significant difference between Ctrl + DMSO, CPZ + 15 μg OZ and CPZ + 30 μg OZ groups. These results suggest that OZ might rescue the loss of oligodendrocytes in the corpus callosum of cuprizone‐induced mice.

### 3.3 OZ rescued the loss of oligodendrocytes through the TAK1 signaling pathway in CPZ‐induced mice

**FIGURE 3:**
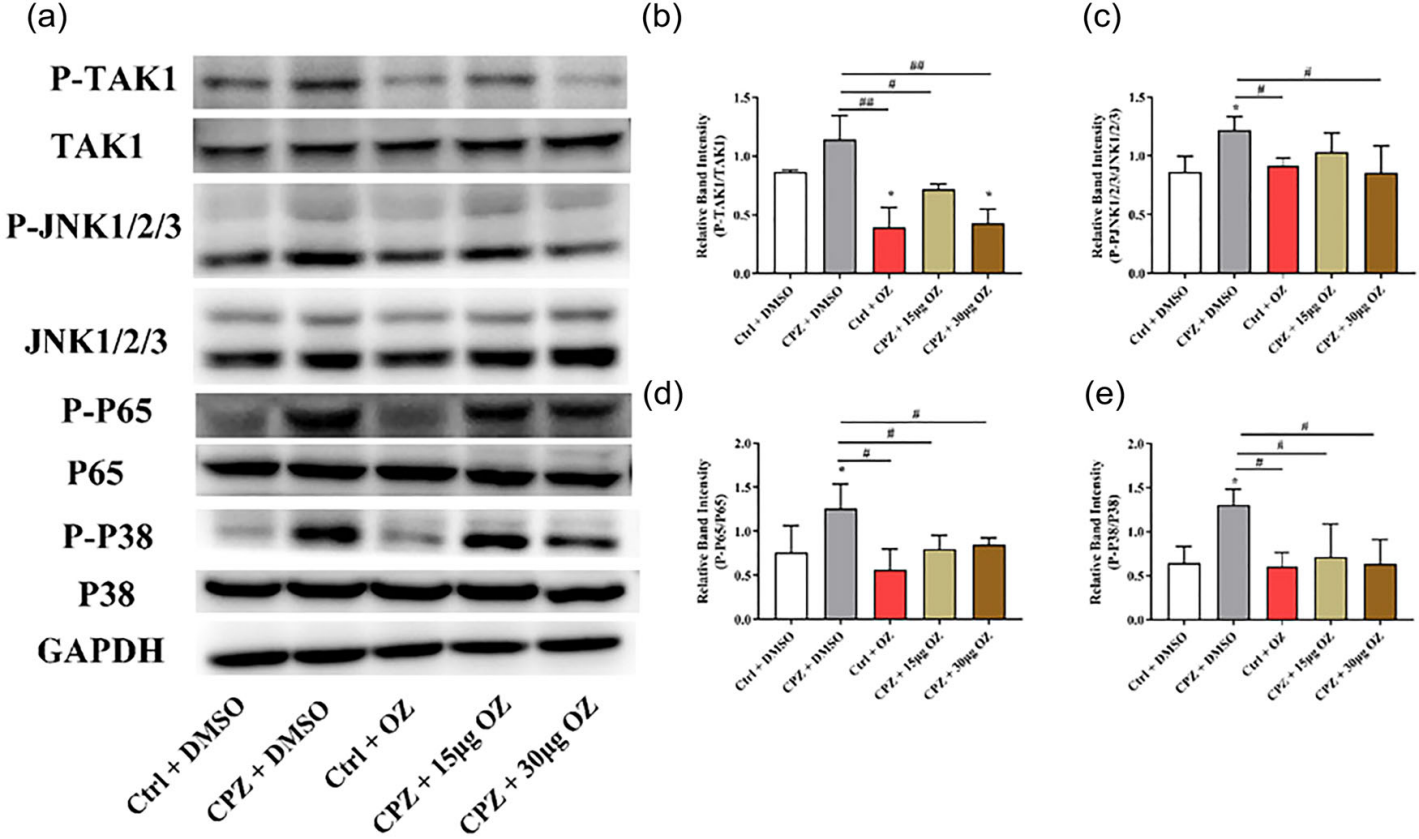
OZ rescued the loss of oligodendrocytes through TAK1 signaling pathway in CPZ‐induced mice. (a–e) Western blots and quantitative analysis showing the expression levels of P‐TAK1, TAK1, P‐JNK1/2/3, JNK1/2/3, P‐p65, p65, P‐p38, and p38 in the corpus callosum in the Ctrl + DMSO, CPZ + DMSO, Ctrl + OZ, CPZ + 15 μg OZ and CPZ + 30 μg OZ groups (*n* = 3). Representative blots are shown from the same sample. Data are presented as the mean ± SEM. ^*^ *p* < .05 vs. the Ctrl + DMSO group. ^##^ *p* < .01, ^#^ *p* < .05 vs. the CPZ + DMSO group. One‐way ANOVA with Tukey's post hoc analysis was used for comparison among multiple groups.

As a selected inhibitor that suppresses TAK1 activation, we analyzed corpus callosum homogenates by Western blotting to measure phosphorylated TAK1 and total TAK1 levels, confirming the influence of OZ administration on TAK1 in the CPZ‐induced demyelinated mouse model. Compared to the Ctrl + DMSO group, CPZ increased the phospho‐TAK1 level, which was decreased after treatment with 15 and 30 μg of OZ (*F* = 17.319, *p* < .001) (Figure 3a and b). This supports the idea that the activation of TAK1 mediates demyelination in CPZ mice and verifies the function of OZ in suppressing TAK1 activation. As a common upstream activator of JNK, p65, and p38 signaling pathways, the inhibition of TAK1 function may have an impact on these downstream signal cascades. To determine the effect of OZ on these downstream signaling cascades in the CPZ mice, we performed Western blotting of corpus callosum homogenates from mouse brains. Compared to the Ctrl + DMSO group, CPZ activated the JNK, p65, and p38 pathways, which are the downstream signaling molecules of TAK1 (Figure 3c–e). These activations were attenuated in the CPZ + 15 μg OZ and CPZ + 30 μg OZ groups (JNK: *F* = 3.081, *p* > .05; p65: *F* = 3.876, *p* < .05; p38: *F* = 4.229, *p* < .05). However, no statistical reduction in JNK phosphorylation was observed between the CPZ + DMSO and CPZ + 15 μg OZ groups. These results suggest that OZ inhibits the activation of JNK, p65, and p38 pathways.

### 3.4 OZ Inhibits the activation of microglia and increases the M2/M1 polarization ratio in CPZ‐induced mice

**FIGURE 4:**
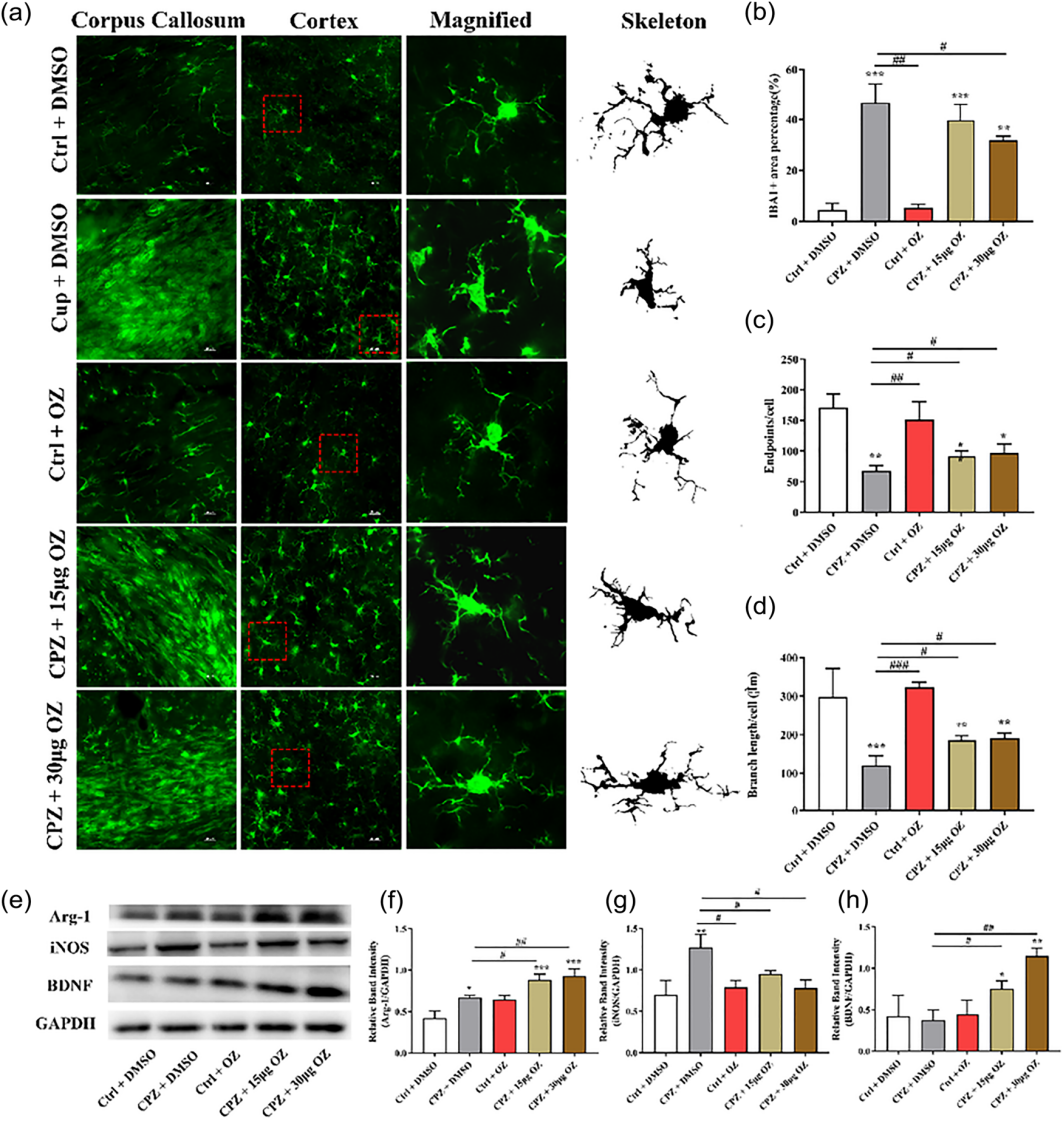
OZ promotes M2 polarization of microglia and reduces M1 polarization in CPZ‐induced mice. (a) Immunofluorescence staining of Iba1‐positive microglia in the Ctrl + DMSO, CPZ + DMSO, Ctrl + OZ, CPZ + 15 μg OZ, and CPZ + 30 μg OZ groups (*n* = 4). Maximum intensity projection of fluorescence images was transformed into binary images and then skeletonized. (b) Quantitative analysis of fluorescence intensity in the corpus callosum in five groups. (c, d) Quantitative analysis of the number of endpoints and process length of Iba1‐positive cells after skeletonization. (e–h) Western blots and quantitative analysis showing the expression levels of Arg‐1, iNOS, and BDNF. Representative blots are shown from the same sample. Data are presented as the mean ± SEM. ^***^ *p* < .001, ^**^ *p* < .01, ^*^ *p* < .05 vs. the Ctrl + DMSO group. ^###^ *p* < .001 ^##^ *p* < .01, ^#^ *p* < .05 vs. the CPZ + DMSO group. One‐way ANOVA with Tukey's post hoc analysis was used for comparison among multiple groups.

To determine the effect of OZ on microglia, we first detected the expression of Iba1, a microglia marker, by immunofluorescence analysis. Compared to the Ctrl + DMSO group, CPZ significantly increased the Iba1+ area percentage in the corpus callosum. Both 15 and 30 μg OZ treatment reduced the Iba1+ area percentage in CPZ‐induced mice (*F* = 53.231, *p* < .001) (Figure 4a and b). To further study the effect of OZ on microglia, we analyzed the morphology of microglia in the cortex. The microglia in the Ctrl + DMSO group exhibited a characteristic static phenotype with fine‐branching morphology. The CPZ + DMSO group showed more Iba1+ cells, which changed to the shape of large and round phagocytes. Additionally, OZ treatment increased the microglia endpoint and branch length of Iba1+ cells in CPZ mice, indicating that microglia were deactivated in the presence of OZ (endpoint: *F* = 17.851, *p* < .001; branch length: *F* = 21.727, *p* < .001) (Figure 4c and d). To investigate the change, we performed Western blotting to distinguish different phenotypes of microglia. Arg‐1 is a marker of M2 microglia/macrophage, while iNOS is a marker of M1 microglia/macrophage. Interestingly, compared with the CPZ + DMSO group, OZ increased the expression of Arg‐1 and decreased the expression of iNOS in the CPZ + 15 μg OZ and CPZ + 30 μg OZ groups (Arg‐1: *F* = 29.328, *p* < .001; iNOS: *F* = 10.707, *p* < .01) (Figure 4e–g). These data indicate that OZ attenuates CPZ‐induced demyelination by suppressing M1 microglia/macrophage polarization and increasing M2 microglia/macrophage polarization. Additionally, compared to the CPZ + DMSO group, OZ increased the expression of brain‐derived neurotrophic factor (BDNF), which can accelerate the remyelination of injured axons (Su et al., 2019) (*F* = 12.850, *p* < .01) (Figure 4h).

### 3.5 The effect of OZ on proinflammatory cytokines and anti‐inflammatory cytokines in CPZ‐induced mice

**FIGURE 5:**
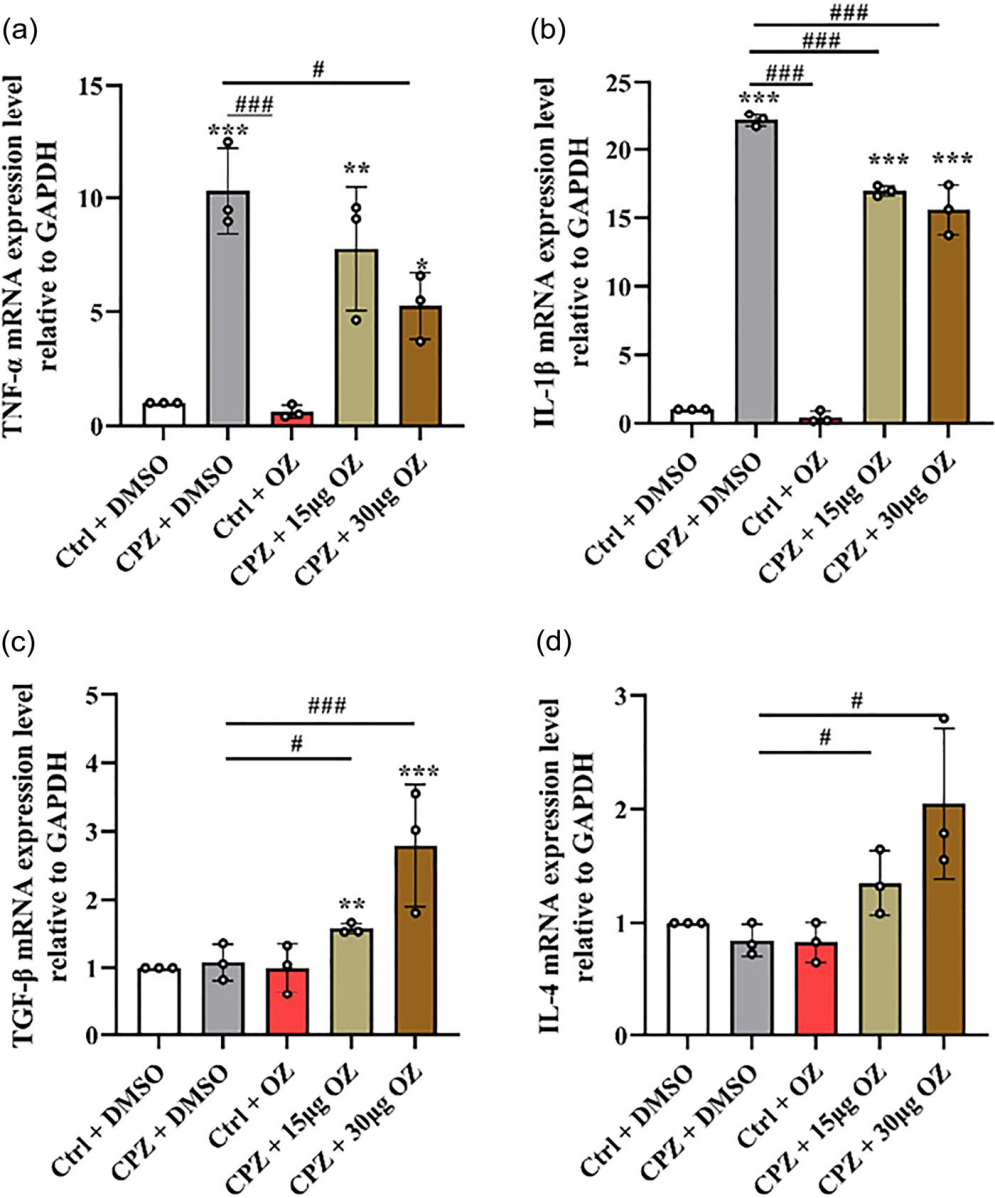
OZ decreased the expression of proinflammatory cytokines genes and increased the expression of anti‐inflammatory cytokines genes in CPZ‐induced Mice. (a–d) The mRNA expression levels of TNF‐α, Il‐1β, TGF‐β and IL‐4 in the cerebral cortex in the Ctrl + DMSO, CPZ + DMSO, Ctrl + OZ, CPZ + 15 μg OZ, and CPZ + 30 μg OZ groups (*n* = 3). Data are presented as the mean ± SEM. ^***^ *p* < .001, ^**^ *p* < .01, ^*^ *p* < .05 vs. the Ctrl + DMSO group. ^###^ *p* < .001, ^##^ *p* < .01, ^#^ *p* < .05 vs. the CPZ + DMSO group. One‐way ANOVA with Tukey's post hoc analysis was used for comparison among multiple groups.

**FIGURE 6:**
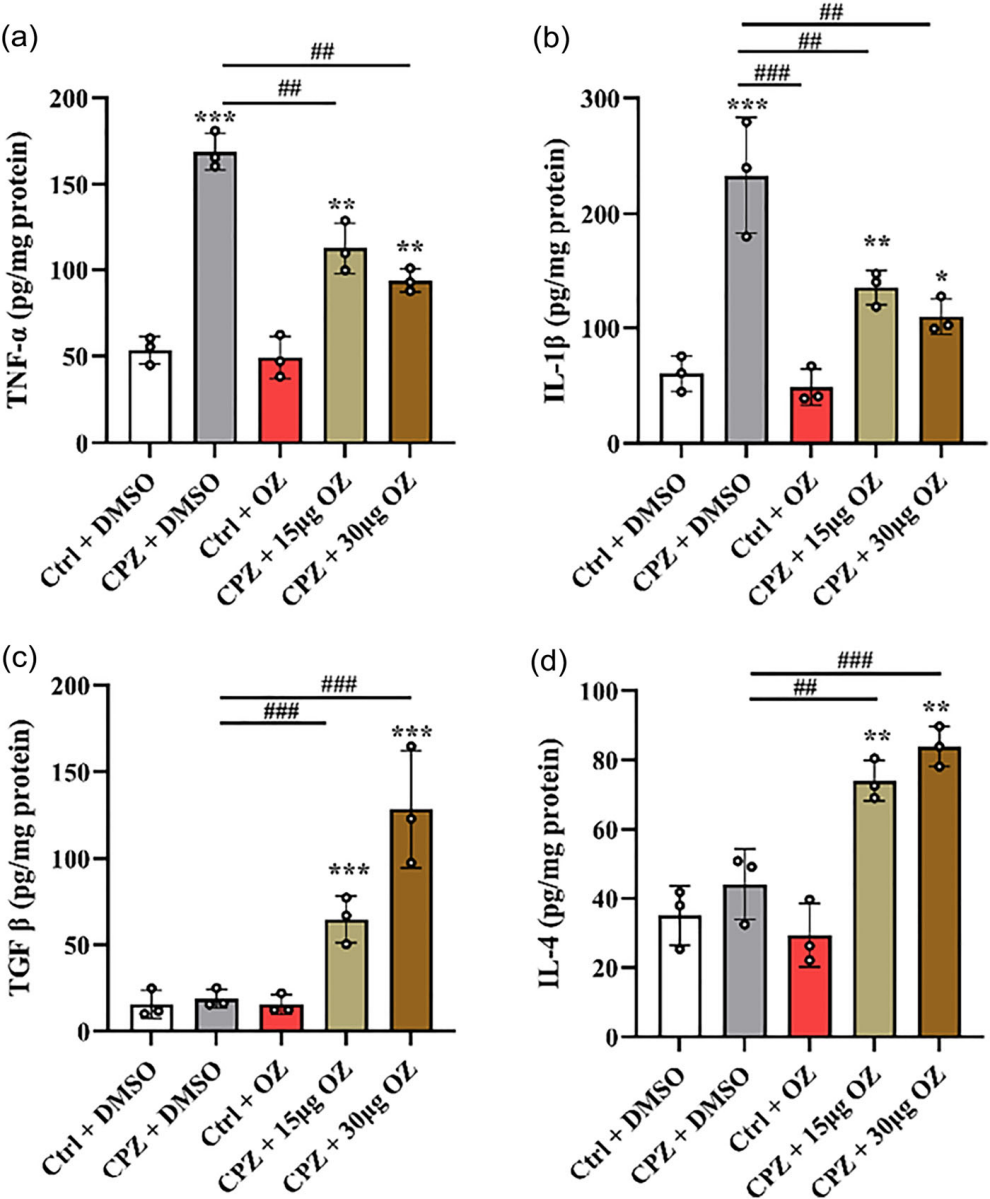
OZ decreased the concentrations of proinflammatory cytokines and increased the concentrations of anti‐inflammatory cytokines in CPZ‐induced Mice. (a–d) The concentrations of TNF‐α, IL‐1β, TGF‐β, and IL‐4 in the cerebral cortex were measured in the Ctrl + DMSO, CPZ + DMSO, Ctrl + OZ, CPZ + 15 μg OZ, and CPZ + 30 μg OZ groups (*n* = 3). Data are presented as the mean ± SEM. ^***^ *p* < .001, ^**^ *p* < .01, ^*^ *p* < .05 vs. the Ctrl + DMSO group. ^###^ *p* < .001, ^##^ *p* < .01, ^#^ *p* < .05 vs. the CPZ + DMSO group. One‐way ANOVA with Tukey's post hoc analysis was used for comparison among multiple groups.

To investigate the impact of OZ on microglia polarization, we conducted qPCR analysis to measure the gene expression levels of proinflammatory and anti‐inflammatory cytokines. In comparison to the Ctrl + DMSO group, the CPZ + DMSO group exhibited significantly increased gene expression levels of proinflammatory cytokines including tumor necrosis factor (TNF)‐α and interleukin (IL)−1β. However, in the CPZ + 15 μg OZ and CPZ + 30 μg OZ groups, the expression of IL‐1β and TNF‐α was noticeably reduced (IL‐1β: *F* = 378.768, *p* < .001; TNF‐α: *F* = 46.414, *p* < .001) (Figure 5a and b). Moreover, the CPZ + 15 μg OZ and CPZ + 30 μg OZ groups demonstrated elevated levels of anti‐inflammatory cytokines such as transforming growth factor (TGF)‐β and IL‐4, compared to the Ctrl + DMSO and CPZ + DMSO groups (TGF‐β: *F* = 8.776, *p* < .01; IL‐4: *F* = 6.863, *p* < .01) (Figure 5c and d). Furthermore, the ELISA results demonstrated that compared to the CPZ + DMSO group, demyelinated mice treated with OZ exhibited a reduction in the levels of the proinflammatory cytokines TNF‐α and IL‐1β, along with an increase in the levels of the anti‐inflammatory cytokines TGF‐β and IL‐4 (TNF‐α: *F* = 60.358, *p* < .001; IL‐1β: *F* = 23.117, *p* < .001; TGF‐β: *F* = 24.818, *p* < .001; IL‐4: *F* = 26.911, *p* < .001) (Figure 6a–d).

## 4 DISCUSSION

Demyelination and remyelination are crucial pathophysiological processes that play a significant role in determining the outcome of MS. These processes occur simultaneously and share several effector molecules. In our current study, we have shown that TAK1 inhibitor OZ acts as an upstream regulator, transforming M1‐polarized microglia into M2 phenotype, rescuing the loss of oligodendrocytes, mediating inflammation, and increasing BDNF expression in CPZ‐induced demyelination mice model.

The CPZ‐induced demyelination animal model is a widely used method to study demyelination diseases similar to those observed in patients with MS (Toomey et al., 2021). CPZ administration leads to extensive demyelination of the corpus callosum and motor coordination disorders in mice. Consistent with previous studies, we found that CPZ treatment resulted in motor dysfunction in mice (Khaledi et al., 2021; Sui et al., 2019). In our study, we administered CPZ orally for 5 weeks to induce acute demyelination in mice. We used OZ, a highly selective TAK1 inhibitor, to inhibit TAK1 activity (Wu et al., 2013). Administration of OZ improves motor dysfunction of demyelination mice, which is consistent with our previous research (Lu et al., 2017). Furthermore, OZ treatment prevented CPZ‐induced myelin loss. Conditional depletion of TAK1 in microglia also significantly reduced CNS inflammation and diminished axonal and myelin damage in a mouse MS model of EAE (Goldmann et al., 2013).

TAK1 plays a crucial role in regulating a diverse array of physiological and pathological cellular processes (Ajibade et al., 2013; Shim et al., 2005). It serves as a key upstream integrator in multiple proinflammatory signaling pathways, orchestrating the synthesis of chemokines, proinflammatory cytokines, and adhesion molecules. Targeting TAK1 production has emerged as a potential therapeutic approach for managing chronic inflammatory disorders, such as autoimmune diseases and cancer (Sakurai, 2012). Once activated, TAK1 activates crucial intracellular kinases, including the p38, JNK, and p65 (Ajibade et al., 2013). These signaling molecules are involved in the pathogenesis of demyelinating diseases (Cohen, 2009; Yadav et al., 2021a). Overactivation of JNK/p38 signaling has been observed in demyelination and neuroinflammation (Singh et al., 2021). Downregulation of c‐JNK/p38MAPK can promote oligodendrocyte regeneration and prevent mitochondrial dysfunction, microglia survival, and neurotransmitter recovery (Yadav et al., 2021b). Activation of p65 in astrocytes and microglia appears to favor demyelination, while its inhibition supports remyelination (Blank & Prinz, 2014). Our results showed that CPZ treatment activated TAK1 and its downstream signaling proteins JNK, p65, and p38. However, OZ treatment diminished the phosphorylation levels of these proteins in CPZ‐treated mice. Therefore, inhibition of TAK1 may be an effective therapy in demyelination and remyelination process.

Remyelination, the process of newly differentiated oligodendrocytes forming myelin sheaths around demyelinated axons, is crucial for recovery from demyelinating diseases. Olig2 is a transcription factor that plays a crucial role in regulating the specification of neurons and glial cells, as well as promoting the differentiation of oligodendrocytes and their precursor cells (Marshall et al., 2005). Previous studies have demonstrated that treatment with CPZ leads to a decrease in the number of oligodendrocytes in the corpus callosum (Ye et al., 2013). However, in comparison to the CPZ + DMSO group, treatment with OZ not only increased the number of CC1^+^‐positive cells but also augmented the number of Olig2^+^‐ cells in CPZ‐treated mice. These findings suggest that OZ treatment can prevent the loss of oligodendrocytes. Additionally, we found that OZ treatment increased the protein expression of BDNF, which enhances the differentiation of oligodendrocyte lineage cells (Saitta et al., 2021). These results suggest that OZ has a neuroprotection on demyelinated mice.

Microglia activation after CPZ administration not only damages myelin regeneration but also leads to progressive demyelination (Marzan et al., 2021). However, microglia can also produce regeneration mediators and play an important role in the phagocytic process (Sen et al., 2022). Microglia with different phenotypes have different functions in demyelinating diseases. M1‐activated microglia/macrophages participate in autoimmune disease damage to CNS by secreting toxic molecules and presenting antigens to cytotoxic lymphocytes (Peng et al., 2019; Tang & Le, 2016). M2‐activated microglia/macrophages are responsible for resolving inflammation and repairing damaged tissues through phagocytosis and secretion of anti‐inflammatory mediators (Aguilera et al., 2018; Praet et al., 2014; Wang et al., 2019). Additionally, M2 microglia/macrophages can drive oligodendrocyte differentiation during CNS remyelination (Miron et al., 2013). Therefore, it is crucial to explore medicines or targets that can transform microglia into M2 phenotype. Our results suggested that OZ reduces microglia activation, increases the expression of Arg‐1 and decreases the expression of iNOS. These suggested that OZ promote transformation of microglia into M2 phenotype. Furthermore, OZ treatment decreases the secretion of proinflammatory cytokine secretion, including TNF‐α and IL‐1β, while increasing the secretion of anti‐inflammatory cytokine secretion, including TGF‐β, IL‐4 in CPZ‐treated mice.

## 5 CONCLUSION

In conclusion, our findings indicate that inhibiting TAK1 function with OZ may have a therapeutic potential in demyelinating diseases. OZ treatment effectively prevents the loss of oligodendrocytes in CPZ‐treated mice, transforms microglia into an M2 phenotype, increases BDNF expression, and decreases the expression of proinflammatory cytokines while increasing anti‐inflammatory cytokines in CPZ‐treated mice. These findings suggest that OZ may be a promising treatment for demyelinating diseases.

## AUTHOR CONTRIBUTIONS

**Shiyu Chen**: Validation; data curation; methodology; software; writing—original draft. **Siyao Liu**: Formal analysis; visualization. **Yalun Huang**: Formal analysis. **Shiwen Huang**: Formal analysis. **Wanzhou Zhang**: Validation. **Huifang Xie**: Conceptualization; methodology; writing—review and editing; supervision; project administration. **Lingli Lu**: Conceptualization; methodology; writing—review and editing; funding acquisition; supervision; project administration.

## CONFLICT OF INTEREST STATEMENT

The authors have no conflicts of interest concerning this work.

### PEER REVIEW

The peer review history for this article is available at https://publons.com/publon/10.1002/brb3.3487.

## Data Availability

The data that support the findings of this study are available on request from the corresponding author.
